# Emerging challenges: clozapine-associated hepatotoxicity in bipolar disorder: a case report

**DOI:** 10.1097/MS9.0000000000002741

**Published:** 2025-01-09

**Authors:** Hassan Omar Hersi, Mohamed Osman Omar Jeele, Bakar Ali Adam, Mohamed Omar Hassan

**Affiliations:** a Department of Internal medicine; b Department of Neurology; c Department of Cardiology, Mogadishu Somali Turkish Training and Research Hospital, Mogadishu, Somalia

**Keywords:** bipolar disorder, clozapine, drug-induced liver injury, hepatotoxicity, Somalia

## Abstract

**Introduction:**

Clozapine can cause major adverse effects, including rare but serious drug-induced liver injury. Understanding how clozapine causes liver injury is crucial for prompt diagnosis and effective management.

**Case presentation:**

We presented a case of a 42-year-old man with bipolar disorder who presented to our emergency department with a complaint of fatigue, nausea, vomiting, and pain in the upper right abdomen for 1 week. The patient was commenced with clozapine 1 month prior. He appeared jaundiced with a slightly swollen abdomen. Blood tests revealed highly elevated liver enzymes (AST 1679 U/L, ALT 1752 U/L) and bilirubin levels (total bilirubin 8.5 mg/dL, direct bilirubin 3.02 mg/dL). Tests for viral hepatitis and autoimmune diseases were negative. Suspecting clozapine-induced liver injury, we stopped the medication and provided supportive care.

**Discussion:**

Clozapine-induced liver injury likely occurs through a variety of pathophysiological pathways that include its metabolism by cytochrome P450 enzymes. Recognizing symptoms like jaundice and abdominal pain early is crucial for diagnosis. Our case reflects similar cases in literature, highlighting the variability in how this condition presents and the importance of prompt intervention to prevent severe consequences. Treatment involves stopping clozapine, supportive care, and closely monitoring the patient’s recovery.

**Conclusion:**

Physicians who prescribe clozapine should make sure that liver function is closely monitored, especially in the initial few months of the medication, and stop it if liver function become more than 3 times of upper limit of normal and patient developed signs and symptoms of liver injury.

## Introduction

Clozapine, an atypical antipsychotic globally used for treatment-resistant schizophrenia, has established outstanding efficacy in treating severe psychiatric disorders. However, its clinical usage is tempered by possible negative effects, namely uncommon yet significant hepatotoxicity. Clozapine-induced liver injury characterizes a complicated clinical difficulty described by differing degrees of hepatic impairment, encompassing from mild transaminase increase to severe hepatitis and fulminant hepatic dysfunction. Understanding the mechanisms underlying clozapine-induced liver injury is significant for prompt identification, treatment, and reduction of its possible life-threatening consequences^[1,2]^.

It is hypothesized that the hepatotoxic effects of clozapine are caused by the metabolic transformation of the drug and ensuing interactions with liver enzymes and pathways. Clozapine undergoes a complete metabolism, principally through the action of cytochrome P450 enzymes. This results in the generation of reactive metabolites, which have the potential to disrupt cellular integrity and calm immune-mediated responses inside the hepatic cells. The distinctive character of clozapine-induced liver injury is brought to light by this diverse pathophysiology, which makes the identification and management of this condition more difficult in clinical settings^[3,4]^.

This case study reports a gripping illustration of clozapine-induced liver injury in a 42-year-old male patient with bipolar disorder, underscoring the clinical manifestations, diagnostic difficulties, and management. By explaining the complexities of clozapine-induced hepatotoxicity, this case study tends to increase attention among physicians relating to the possible negative effects associated with clozapine therapy and the significance of vigilant observation and prompt management in decreasing negative effects.

## Case report

A 42-year-Old male come to our Emergency Department with complaint of fatigue, nausea, vomiting and right upper quadrant pain for 1 week. His physical examinations revealed jaundice, drowsiness with early arousable, abdomen mildly distended and soft, and no stigma of chronic liver disease. Cardiopulmonary examinations were unremarkable. Vital signs were within normal limits. He had a past medical history of bipolar disorder with multiple anti-psychotic medications for last 2 years with poor medical follow-up. His previous medications which he stopped by himself included risperidone and fluoxetine. His psychiatry doctor commenced clozapine 1 month ago and his dose was titrated up to 300 mg per day. No history of concomitant liver disease, other medications, surgical history, smoking, alcohol, and substance use.

Laboratory investigations revealed AST 1679 U/L, ALT 1752 U/L, total bilirubin’s 8.5 mg/dL, direct bilirubin’s 3.02 mg/dL, and INR 1.9 (Table 1). Other laboratory test including full blood count, renal function test and thyroid function test was normal. Vitamin B12, folic acid, and iron study was normal. No prior laboratory records were available. Results for viral serology including hepatitis A, B, and C were negative. An autoimmune analysis was negative for anti-mitochondrial antibody, antinuclear antibody, and anti-smooth muscle antibody. Abdominal ultrasound was performed and came back negative.

**Table 1:** Liver function tests of the patient ranging from day 1 to 2 weeks

Parameters	Normal range	Day 1	Day 3	Day 5	Day 14
AST	0–35 U/L	1679	290	168	32
ALT	0–45 U/L	1752	833	636	43
T. BILURIBIN	0.1–1.2 mg/dL	8.55	3.51	2.55	1.17
D. BILURIBIN	0.01–0.4 mg/dL	3.02	2.19	1.61	0.35

**Figure 1:**
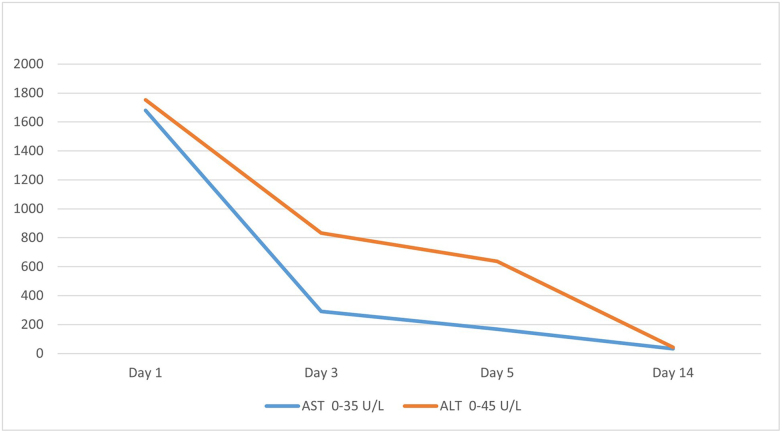
Progression of aminotransferase levels of the patient.

With regard to his history, clozapine toxicity was suspected and patient was admitted to hospital along with supportive care. Clozapine immediately was stopped. After 3 days in the hospital, his liver enzyme levels were dropped significantly; AST 290 U/L and ALT 833 U/L (Fig. 1). During the fifth day of hospitalization, the patients’ biochemical results improved drastically with AST 168 U/L and ALT of 636 U/L and also his overall clinical condition improved and he was discharged from the hospital. He returns to hospital 2 weeks later and his liver enzyme levels shows normal range.

## Discussion

Given its greater efficacy, clozapine, which is a strong atypical antipsychotic, is frequently utilized in the treatment of schizophrenia and bipolar disorder that is resistant to other treatments^[5]^. On the other hand, its utilization is linked to a number of negative effects, the most significant of which is hepatotoxicity, which is uncommon but substantial. Our case report of a 42-year-old male with bipolar disorder is reported here as an illustration of a compelling incidence of drug-induced liver damage caused by clozapine. This case study emphasizes the complexity connected with diagnosis, therapy, and potential consequences associated with the ailment.

The manifestation of clozapine-induced liver injury occurs through a variety of pathophysiological pathways that include its metabolism by cytochrome P450 enzymes. These enzymes produce reactive compounds that are capable of inducing cellular damage and immune-mediated reactions inside hepatic tissue. This case demonstrates the significance of spotting early clinical signs such as fatigue, nausea, vomiting, discomfort in the right upper quadrant, and jaundice. These symptoms prompted an immediate investigation, which ultimately led to the diagnosis of hepatotoxicity^[6]^.

The challenges and outcomes that were observed in our patient are apparent in other cases that have been published in the literature. For instance, a case that was reported by Ha *et al* showed a 39-year-old male patient with schizophrenia who suffered from acute liver injury after commencing treatment with clozapine. The patient presented with symptoms that were comparable to those of our patient, and the injury resolved quickly after stopping the drug^[7]^. In a similar manner, Bjornsson *et al* conducted research in which they examined several cases of liver damage caused by clozapine. The primary focus of their investigation was to highlight the heterogeneity in clinical presentation and the need of early intervention in order to prevent serious consequences^[8]^.

Arndtz *et al* in 2020 described a patient in Italy who had acute hepatitis 3 weeks after initiating clozapine and demonstrated a similar pattern of quickly enzyme rise and normalization after discontinuing the drug^[9]^. Another instance from Japan, detailed by Saito *et al* (2019), was a patient with transaminitis 4 weeks into clozapine therapy who demonstrated complete biochemical remission following termination^[10]^. Similarly, De Leon *et al* in 2020 described a case in the United States in which a patient with no underlying liver illness saw a large increase in AST and ALT 1 month after starting clozapine^[11]^.

In order to effectively manage clozapine-induced hepatic ischemia-induced liver injury, it is necessary to promptly discontinue the offending medication, provide supportive care, and closely monitor liver function tests. These case reports collectively suggest that clozapine-induced liver injury is reversible if identified early, with complete recovery typically observed within weeks of stopping the drug. The patient in our study had a considerable improvement in liver tests and clinical symptoms after a few days of discontinuing clozapine treatment, emphasizing the fact that this negative effect can be reversed with appropriate intervention similar with previous case reports^[7,8]^.

It is also notable that comparable instances that were described by Andrade *et al* and Sgro *et al* had similar clinical course and outcome as our case. This highlights the critical role that early detection and management play in reducing the severity of clozapine-induced hepatotoxicity^[1,12]^.

## Conclusion

The need of constant observation and immediate act to maximize patient outcomes is underlined in this case study. In light of this, physicians who prescribe clozapine should make sure that liver function is closely monitored, especially in the initial few months of the medication, and stop it if liver function become more than 3 times of upper limit of normal and patient developed signs and symptoms of liver injury.

Additional research is necessary to clarify the fundamental causes and risk factors related with clozapine-induced liver injury, thus guiding strategies for better patient care and safer prescribing guidelines.

## Data Availability

Data are available from the corresponding authors if requested.
